# Taxonomic Impediment for Conservation: The Case of Bees in an Undersampled Tropical Mid-Elevation Site, San Martín, Peru

**DOI:** 10.3390/insects15070544

**Published:** 2024-07-18

**Authors:** Claus Rasmussen, Evelyn Sánchez

**Affiliations:** 1 Department of Agroecology, Aarhus University, 4200 Slagelse, Denmark; 2 Facultad de Ciencias Biológicas y Veterinarias, Universidad Científica del Sur, Lima, Peru; 3 Departamento de Entomología, Museo de Historia Natural, Universidad Nacional Mayor de San Marcos, Lima, Peru

**Keywords:** Amazon basin, Alto Mayo, diversity, pollinators, ecology

## Abstract

**Simple Summary:**

Knowledge about the pollinator species as required to both value and conserve them. This study explores some of the difficulties when assessing a tropical fauna, with a large proportion left unidentified, although generalities about their biology can still be retrieved based on knowledge of the genera and related species.

**Abstract:**

In this first field survey of an entire bee fauna for any part of Peru, we report a total of 1796 bees belonging to 181 species or morphospecies in four families. The taxonomic impediment was pronounced with only 80 species of 181 that could be named. With such a high proportion of undetermined species, it is not possible to adequately compare pollinator communities across different studies, assess historical changes or analyze endemism patterns to document ecology, behavior and evolution of the species and genera. This information is required to provide a sound basis for policymakers to protect habitats for the conservation of native pollinators.

## 1. Introduction

Pollination is vital for reproduction of angiosperms in all terrestrial ecosystems and is considered a fundamental process for the sustainability of flora and fauna [1]. About 88% of the flowering plant species are pollinated by animals [2] and more than 75% of the main global food crops depend on pollinators [3]. Insects, and mainly the bees, are considered primary pollinators of most agricultural crops and wild plants [4]. The ecosystem services provided by these pollinators have a global economic value of approximately 153 billion Euros [5]. An additional underestimated value in agricultural production may come from cases where inadequate pollination causes inferior quality products or fruit abortion [6].

**Figure 1:**
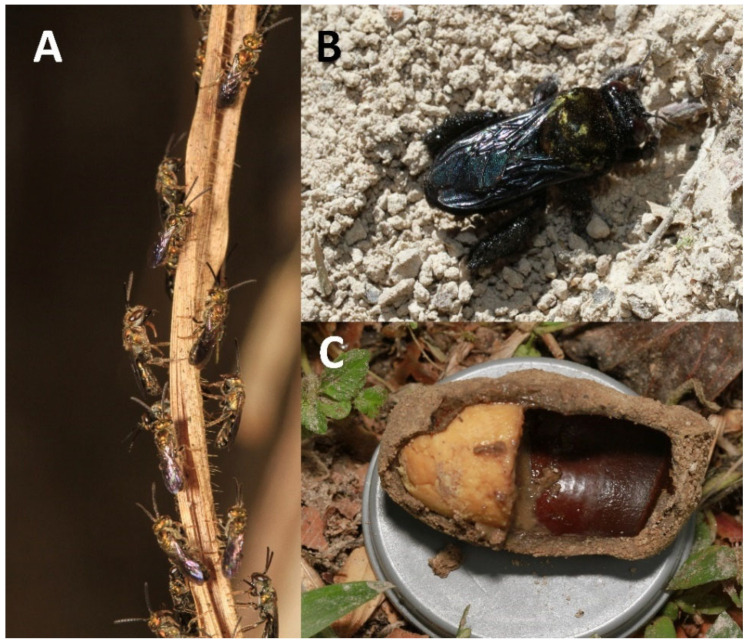
(**A**) In the humid Andes in South America, some bees (*Augochlora*, *Augochloropsis*, *Neocorynura* a.o.) are diverse and largely impossible to identify. (**B**) Female of *Centris* (*Melacentris*) from RA, including the nest cell with provision (**C**). Biology is unknown for species that are not identifiable. This specimen collected during this study was later described as *C. fuscolineata*.

Reported declines in native bee populations [7,8] are, therefore, of public concern, and baseline data on populations are required for monitoring apparent pollinator declines. Although many general insect surveys (including captures of bees) have been carried out in Peru, there is a paucity of published bee checklists. Besides the early simple country lists by Moure [9,10] and the more recent compilation of Neotropical literature records [11], lists enumerating the entire bee fauna are not available for any locality in Peru. Regional records can be extracted from [11], and these data, with updates, are also mapped on Discover Life [12], but even regional records are fragmentary and some published records have been attributed to the wrong region. An updated country list will provide some new records, but also point to lack of knowledge for many taxonomic groups and regions of the country (Rasmussen & Ascher, in preparation). This might be due to the taxonomic challenge of identifying and reporting a bee fauna as poorly characterized as that of the tropical Andean countries (Figure 1).

Local surveys have often been limited to either taxonomic groups, in particular orchid bees or Euglossini [13,14,15,16,17,18,19], stingless bees or Meliponini [20,21,22] and bumble bees or Bombini [23]. Peruvian bee taxa have frequently been described or recorded in the context of taxonomic revisions of Peruvian material [24,25,26,27,28,29,30,31,32,33,34,35,36,37,38,39,40,41,42], with many further records found in global revisions (e.g., [43,44,45]), or in the limited number of ecological studies or behavioral observations [46,47,48,49,50,51,52]. Moure, Urban and Melo’s [11] document 592 described and reported species of bees from Peru, with an estimated potential of 946 species in total. 

**Figure 2:**
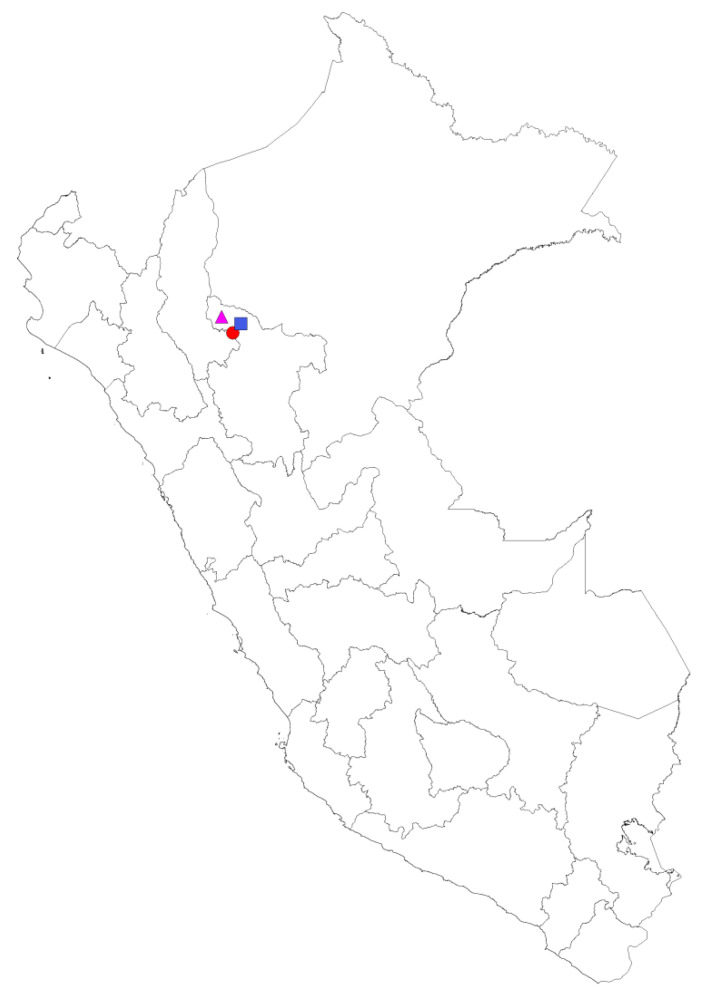
A map of Peru with the three collecting sites indicated. Río Negro (RN, red circle), Ríos Aguas Claras-Amangay (RA, pink triangle) and Tingana (TI, blue square).

**Figure 3:**
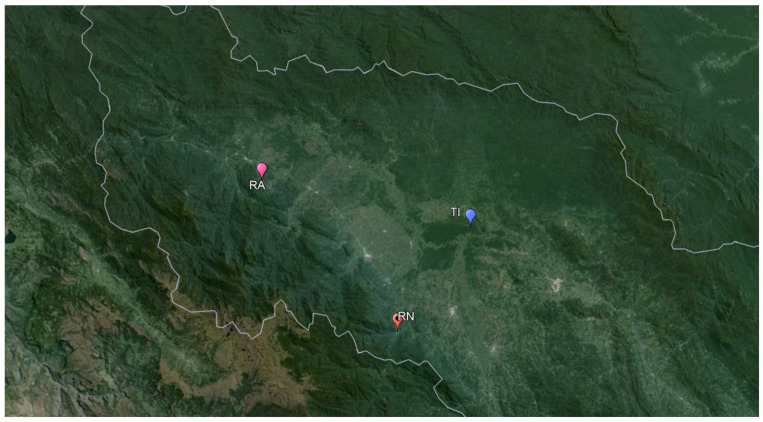
Google Earth map of the three collecting sites indicated (Río Negro [RN], Ríos Aguas Claras-Amangay [RA] and Tingana [TI]).

The Alto Mayo subbasin, region of San Martín, northeastern Peru (Figure 2 and Figure 3), is interesting due to the location in a transition zone between lowland rainforest and lower montane rainforest, or cloud forest. For some well-known vertebrate groups, such as birds, these humid mid-elevation areas are known to contain the highest species richness and endemism [53], in particular near historic population centers [54], such as the archeologically rich Alto Mayo valley.

The purpose of this study is to provide the first Peruvian local list for bee species based on a recent inventory of the bee-fauna of Alto Mayo in northeastern Peru. Specifically, we have chosen three close localities, which include both protected natural habitats and farmland in Alto Mayo, which will provide the following: firstly, assessment of the proportions of different supra-specific groups (families, genera and subgenera) found in a Neotropical mid-elevational site; a quality check of the taxonomic identification literature, or baseline, with respect to completeness for studies at mid-elevation Neotropical sites; brief comparison among the three sites, in particular, in relation to bee nesting biology; a documentation and first step towards identifying the bee fauna in Alto Mayo and Peru, of importance for subsequent monitoring of pollinators in the region. 

The data will be compared to published records of bees from the rest of the region of San Martín to emphasize any bias in published references. Such information will highlight the novelty of the present study and identify unresolved taxonomic impediments that remain a severe hurdle for assessing conservation status, studies of biodiversity and studies of primary agricultural pollinators in the region. 

## 2. Materials and Methods

### 2.1. Study Sites

The study was conducted in three communities near Río Mayo of the Alto Mayo subbasin, between Rioja and Naranjos, in the region of San Martín, northeastern Peru. All three sites had significant coffee production and were selected based on an evaluation of access and presence of areas of conservation that could work as a reserve for natural pollinators and vegetation. From each of the three sites, we would explore the surroundings on foot, looking for and sampling bees. The three sites were: 

Zona de Conservación y Recuperación de Ecosistemas (ZoCRE) Naciente Río Negro, centro poblado Naciente del Río Negro [RN], province of Rioja (6.0854° S, 77.2715° W, 884 m), surveyed 17, 21–23 and 27–29 May 2015 (4 people, 7 days). Río Negro is a community with approximately 287 families and 477 hectares of coffee in the surroundings.

ZoCRE Naciente de los Ríos Aguas Claras-Amangay [RA] (Villa Rica), province of Rioja (5.7838° S, 77.4807° W, 930 m), surveyed 17–20 and 24–25 May 2015 (4 people, 6 days). Ríos Aguas Claras-Amangay is a community with approximately 40 families and 164 hectares of coffee in the surroundings. Both RN and RA are on the orographic left side of the Alto Mayo subbasin or valley. Both are close to running waters, terrain, slopes and trails penetrating nearby protected areas.

ZoCRE Humedal del Alto Mayo sector Tingana [TI] on Río Abisado, province of Moyobamba (5.9157° S, 77.1153° W, 813 m), surveyed 30 May–2 June 2015 (4 people, 4 days). TI is an association with seven families belonging to the Asociación de Conservación de Aguajales y Renacales del Alto Mayo (ADECARAM). The Tingana community combine coffee production with ecotourism, as an activity that promotes conservation and the active participation of the local community, in a flooded Várzea forest. TI is on the orographic right side of the valley and within an aquatic ecosystem partially navigable and accessible by canoes.

### 2.2. Sampling

Approximately eight hours each day were spent in the field (from 8:00 h to 16:00 h) by four people searching for nests, flowers, salt licks and other attractive sites for sweeping and collecting bees. We searched both in wild flowers and in flowering crops within agricultural fields, from flowers of “achiote” (*Bixa orellana*, Bixaceae), “café” (*Coffea arabica*, Rubiaceae), “frijol de palo” (*Cajanus cajan*, Fabaceae), “limón” (*Citrus limon*, Rutaceae), “plátano” (*Musa* sp., Musaceae) and “yuca” (*Manihot esculenta*, Euphorbiaceae), amongst others. In addition to active searching, we ran two malaise traps, one fly catcher or fly trap baited with fermented fish, sprayed honey baits (1:1 honey and water) and placed 28 yellow pan traps with water and soap in the surroundings. Pan traps were emptied daily, whereas malaise traps were placed for the period we were at each of the three sites. Honey baits were often sprayed near the orchid bee baits and swept when bees appeared. Orchid bees, except a few from orchids or nests, were collected using chemical baits. At each site, nine different scented baits were placed ca. 1 m apart from each other at about 1.5 m above the ground. These baits were made of cotton wadding soaked with one the following substances, known or believed to be attractive to orchid bees: benzyl acetate, 1,8-cineole, p-cresol acetate, eugenol, methyl benzoate, methyl trans-cinnamate, methyl salicylate, skatole and vanillin. Baits with cineole, the most volatile compound, were recharged every hour. All bees arriving on the baits during the sampling period were collected, if possible, with insect nets and killed with ethyl acetate and pinned and properly labeled for subsequent identification. As the focus initially was producing raw species lists, labelling of collection date was completed without noting trapping method nor the full period, i.e., Río Negro, 17, 21–23 and 27–29 May 2015; Naciente de los Ríos Aguas Claras-Amangay, 17–20 and 24–25 May 2015; and Tingana, 30 May–2 June 2015. Thus, it was not possible to produce species accumulation curves for the three sites or compare trapping methods.

### 2.3. Identification

The samples were processed and sorted into morphospecies by Evelyn Sánchez and deposited in Museo de Historia Natural, Lima, Peru. From each morphospecies, a series of specimens was compared to reference collections at Museo de Historia Natural (by Evelyn Sánchez) and Claus Rasmussen Collection, Aarhus University, Denmark (by Claus Rasmussen) for identification of most species. In addition, relevant taxonomic literature was surveyed for each of the encountered genera (as listed in e.g., [11]). Assistance was received from various experts, as listed in the acknowledgments section. Unidentified species (marked as “sp.”) are considered impossible to identify at present without engaging in a taxonomic revision of the genera. Confirmed new species will eventually be described by specialists and are reported as “sp. Nov.”. For unidentified species, an attempt was made to match sexes. Therefore, the same species should not be represented twice in the species list by each of the sexes, although it might have failed for some of the more diverse genera. 

### 2.4. Data Analysis

In addition to the species richness, we also report the α-diversity indices of Shannon–Wiener (H′ = −Σ p_i_ ln (p_i_), where p_i_ is the proportion of total number of species made up of the *i*th species) [55]. To test how equally abundant species were in each of the three sites, an evenness measure (E = H′/ln (S), where S is the species richness) was calculated. Lastly, we calculated the indices of Jaccard (C_j_ = a/(a − B + C), where a is the total number of species found in both sites, and B and C are total number of species in each of the two samples) and Sørensen (C_s_ = 2_j_N/(N_a_ + N_b_), where N_a_ and N_b_ are the total number of individuals in each of site a and b, and 2_j_N is the sum of the lower of the two abundances for species found in both sites). These two provide a measure of the pairwise similarity between sites; a higher value indicates more similar sites. The β-diversity index is found by subtracting either of the two indices from 1 (Magurran 2004). Nesting biology for all genera were recorded according to [56]. This includes the categories cleptoparasitic (cp), ground nesting (g), stick or tree cavity nesting (s) or other substrate (o) nesting species. Some genera have representative species that will include multiple categories and are, therefore, recorded as a combination of, for example, ground nesting, stick and tree cavity nesting species (g,s). For comparative purposes, relative summary statistics on the nesting biology were made both on total species and total specimens for each site and visualized in a radar diagram to identify the differences in nesting habitats.

## 3. Results

**Figure 4:**
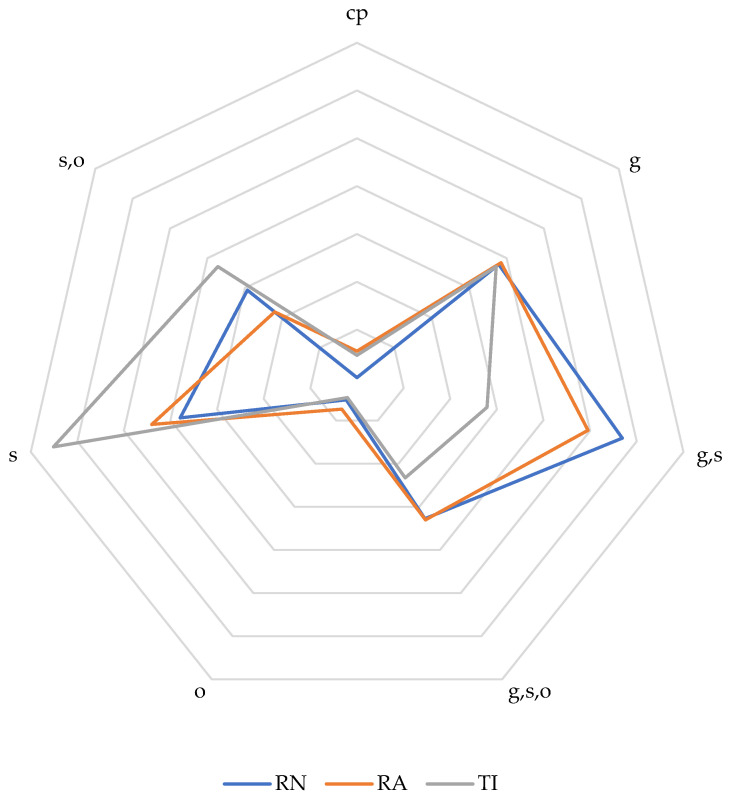
Radar diagram of relative nest site preference for the bee species in the three collecting sites (Río Negro-RN, Ríos Aguas Claras-Amangay-RA and Tingana-TI). The different categories include cleptoparasitic species (cp), ground nesting (g), stick or tree cavity nesting (s) or other substrate (o) nesting species. Combined categories were “g,s” for genera with both ground nesting and stick or tree cavity nesting species; “g,s,o,” ground, stick and other; “s,o” stick and other. For details, see text.

In total, 1796 bees belonging to 181 species or morphospecies in four of the five families found regionally (the family Andrenidae was not encountered) were collected from all three sites in 2015 (Table 1). As expected, the taxonomic impediment was pronounced and only 80 species of 181 could be named. The remaining morphospecies could not be identified at the time using available reference collections or published revisions. Only in the family Apidae could most of the species be identified to species level, except for species in the genus *Ceratina* and the small-to-minute stingless bees of the genera *Plebeia* and *Trigonisca*. The three sites compared poorly overall regarding taxonomic diversity or abundance (Table 2). The most similar sites were Río Negro and Ríos Aguas Claras-Amangay (Cj, 0.25; Cs, 0.56) and the least similar were Río Negro and Tingana (Cj, 0.15; Cs, 0.29). Collections from Alto Mayo are compared to taxa previously reported from San Martín (Table 3). These reports are from localities within the same greater region but made with a different focus and are not directly comparable. However, it is noted that of these three groups, 27 of 61 species are not reported from previous regional studies in San Martín focusing on those specific taxonomic groups. Most of the remaining Apidae species are unreported for San Martín and all the 88 species and morphospecies of Colletidae, Halictidae and Megachilidae are previously unreported from San Martín. Ground or stick nesting was the most frequent nesting site for bees from RN and RA (28% and 25%, respectively), whereas it was stick for TI bees (33%) (Figure 4). 

## 4. Discussion

To our knowledge, this is the first published field survey of an entire bee fauna for any part of Peru. The Alto Mayo subbasin of San Martín in Peru hosts both typical and species-rich humid montane-favoring genera, like *Neocorynura,* and species-rich typical lowland diverse groups, like stingless bees and orchid bees [56]. Therefore, the area represents a novel opportunity for determining the diversity in a potential mega-diverse area consisting of two globally important faunal elements, lowlands and humid montane forests. As expected from the location in a transition zone, we encountered a highly diverse community of bees, much higher than studies from higher altitudes of eastern slopes of southern Ecuador ([57]; 51 spp.) or a literature survey of bees recorded from above 2500 m in Peru ([58]; 67 spp.). Evidently there was a strong element of lowland rainforest species, giving a high richness to the Alto Mayo subbasin, with its mixture of faunas associated with different elevational zones. Identifying the floral hosts of the bees was beyond the scope of the study. However, some relevant observations were made, e.g., that most *Xylocopa* and *Megachile* species collected were visiting flowers of *Cajanus cajan,* whereas most *Epicharis* species collected were visiting flowers from *Bixa orellana*.

### 4.1. Site Differences

From all three sites, we encountered two native species of honey-producing *Melipona* bees. Members of this genus are generally considered to be in decline [59,60] and adversely affected by loss of natural habitat [61], in particular of large trees used for nesting. These *Melipona* were, based on the collecting sites and interviews with the residents, most likely recruited from nests located in the surrounding forests. The abundant natural vegetation on slopes adjacent to Río Negro and Ríos Aguas Claras-Amangay, or across the river Abisado from Tingana, evidently supported this richness of native pollinators. While Río Negro yielded the highest abundance, the diversity was only slightly higher than Ríos Aguas Claras-Amangay. Tingana had fewer bees and, consequently, also less reported diversity. Tingana also had partly flooded Várzea forests and, therefore, difficult to efficiently search for bee nests or aggregations. Curiously, Tingana also had more stick nesting species than ground nesting species, probably a result of the partial and seasonal flooding of the terrain. In addition, only four days were spent collecting in Tingana, whereas we collected for seven and six days in the other two sites. 

### 4.2. Corbiculate Bees

The Alto Mayo sampling included the two bumble bee (*Bombus*) species expected from lowland Peruvian rainforests and the transition zone from montane to lowland rainforest, namely *Bombus transversalis* and *B. pauloensis* [23]. In addition, the Río Negro samples also included a single worker of the much rarer submontane forest species *B. melaleucus,* which is never found in lowland forests (Rasmussen, pers. obs.). Orchid bees and stingless bees are less common at higher altitudes than in lowland tropical forests [58] and we, therefore, expected the diversity to be less than in lowland sites or sites close to lowland forests. In particular, we found orchid bees (*Euglossa*, *Eulaema*, *Exaerete*) to be less abundant and less diverse than at lower altitudes in San Martín (Table 3), yet report new regional records, such as *Euglossa heterosticta,* found nesting in Tingana [62].

Similarly fewer stingless bees were reported than closer to lowland rainforests. Most of the species were either previously reported from San Martín (Table 3) or had been collected elsewhere in San Martín (Rasmussen, unpublished records). The only exception to this, a species not encountered anywhere in Peru at lower altitude, was *Melipona nigrescens* (identification based on reference specimens from a colony kept by Rasmussen and identified by the late J.M.F. Camargo), so far only described and reported from restricted montane rainforests of Colombia above 2500 m elevation [63]. Detailed comparative studies of morphology and DNA should compare the Colombian and Peruvian bees to establish the identity. *Melipona eburnea* was the other species of *Melipona* occurring in Alto Mayo. This is a common and widespread species in the western Amazonia, where it is frequently used for stingless bee keeping or meliponiculture [49,64]. Honey bees, *Apis mellifera*, were recorded from all three sites but were only occasionally abundant, such as in the flowers of *Baccharis* (Asteraceae) in Ríos Aguas Claras-Amangay. They did otherwise not appear abundant and dominant in the vegetation. They were rather local, likely reflecting the recent introduction of apiculture to the region.

### 4.3. Taxonomic Impediment

Addressing the lack of suitable taxonomic references for identification of more than half of the collected taxa is critical for advancing our knowledge of native bees and pollinators in the tropics (see also [65]). Without a proper name, it is not possible to compare communities across different studies, nor is it possible to assess any historical changes in the local bee fauna or to analyze genuine endemism patterns and, thus, document ecology, behavior and evolution of the species and genera. This information is required to provide a sound basis for policymakers to protect habitats for the conservation of native pollinators. The three genera with the largest number of undetermined species are all members of the halictine tribe, Augochlorini. *Neocorynura*, *Augochlora* and *Augochloropsis* have a large number of described species from the Neotropical region, respectively 78, 120 and 143 [11], and an unknown but potentially very large number of undescribed forms as well. Although there are many available names, applying these to newly collected specimens is often impossible without access to primary type series, which often prove to be composites of different taxa and may be dispersed across museums in different countries or continents [66]. This is largely due to inadequate descriptions of species named a century ago or more [67]. As pointed out by Rasmussen [68], a large proportion of the known bee species were described by relatively few European workers and using outdated species concepts relying on coloration or similar. The name-bearing taxa for these above species are mostly stored away in European museums, without yet being fully digitized or otherwise made available to the public in a manner that would facilitate identification and provide global access to information. None of the 27, 15 and 13 species, respectively, collected of these three genera in our study could be convincingly identified based on the published literature. The large number of names available for these genera also impedes the description of new species in such genera, as their novelty cannot be demonstrated without consultation of many relevant types.

### 4.4. Data Issues

Our study only spanned 17 days with four collectors in one season (end of wet season). Surveying throughout the year, including drier and wetter seasons, would likely add many additional species, including known seasonally restricted species, like members of the genus *Eufriesea* [56]. Abundance measures taken for highly eusocial bees, such as stingless bees and honey bees (*A. mellifera*), in the present study are likely strongly biased. Bees from these colonies will recruit foragers to rewarding sites and, even if rare with respect to number of colonies, may, therefore, appear to be locally very common if many workers from one or a few nests are recruited to a specific flower or other food source. The eusocial bees were also collected from nests, but we limited most of the collecting from such places to less than 10 specimens, although some of the aggressive species were collected in higher numbers. This will again affect the abundance. We did not record floral interactions or trapping techniques for the species lists, which prevents us from further analyzing interactions and trapping performance [69], something which should be encouraged for future surveys.

## 5. Conclusions

The high proportion of undetermined species of Halictidae, the most numerous taxa in our study, is an underappreciated fact that emphasizes the continued need for taxonomic support, even for economically important groups, such as the pollinating bees. Further studies should focus on diverse seasonality and different altitudinal zones to determine the relative proportion of the different groups of bees and the diversity maxima of these.

## Figures and Tables

**Table 1:** Species list, species richness (S) and number of specimens (abundance) of each bee species collected at three sites in Alto Mayo, San Martín, Peru. RN: Río Negro, RA: Ríos Aguas Claras-Amangay, TI: Tingana. Stingless bees are indicated with an asterisk. Nest column indicates whether the genus or subgenus is recorded by [56] as a cleptoparasitic species (cp), ground nesting (g), stick or tree cavity nesting (s) or other substrate (o) nesting species.

	Nest	RN	RA	TI	Total
Apidae (S = 93)		551	456	204	1211
*Apis mellifera*	s,o	8	4	5	17
*Bombus* (*Fervidobombus*) *pauloensis*	g		2		2
*Bombus* (*Fervidobombus*) *transversalis*	g			2	2
*Bombus* (*Robustobombus*) *melaleucus*	g	1			1
*Centris* (*Heterocentris*) cf. *Terminata*	s		1	2	3
*Centris* (*Heterocentris*) *chera*	s			2	2
*Centris* (*Heterocentris*) *simplex*	s		1		1
*Centris* (*Heterocentris*) *terminata*	s		1	2	3
*Centris* (*Melacentris*) sp. Nov.	g		8	1	9
*Centris* (*Xanthemisia*) *bicolor*	g,s	1			1
*Cephalotrigona* sp. Nov. *	s	1		15	16
*Ceratina* (*Ceratinula*) sp. 1	s	1			1
*Ceratina* (*Ceratinula*) sp. 2	s	2	2		4
*Ceratina* (*Ceratinula*) sp. 3	s	3	1		4
*Ceratina* (*Ceratinula*) sp. 4	s		1		1
*Ceratina* (*Ceratinula*) sp. 5	s	5	1	1	7
*Ceratina* (*Crewella*) sp. 1	s			8	8
*Ceratina* (*Crewella*) sp. 2	s		2	1	3
*Ceratina* (*Crewella*) sp. 3	s	3	2		5
*Epicharis* (*Epicharana*) *rustica*	g	2			2
*Epicharis* (*Epicharoides*) *albofasciata*	g		1		1
*Epicharis* (*Hoplepicharis*) *affinis*	g	2			2
*Euglossa allosticta*	s,o	3			3
*Euglossa amazonica*	s,o	19	4		23
*Euglossa apiformis*	s,o	1			1
*Euglossa augaspis*	s,o		2		2
*Euglossa heterosticta*	s,o	1	3	6	10
*Euglossa ignita*	s,o	3	22	17	42
*Euglossa imperialis*	s,o	2	2		4
*Euglossa ioprosopa*	s,o	8			8
*Euglossa maculilabris*	s,o	14			14
*Euglossa modestior*	s,o	6	2	1	9
*Euglossa orellana*	s,o	5	14		19
*Euglossa* sp. 1	s,o		1		1
*Euglossa* sp. 2	s,o	1			1
*Euglossa viridifrons*	s,o			1	1
*Eulaema marcii*	s,o	2		3	5
*Eulaema mocsaryi*	s,o		2		2
*Eulaema nigrita*	s,o	1	1	1	3
*Eulaema peruviana*	s,o	3			3
*Eulaema tenuifasciata*	s,o	1			1
*Exaerete smaragdina*	cp		1		1
Geotrigona fulvohirta *	g	2	10		12
*Lophopedia flava*	s	8			8
*Lophopedia haeckli*	s	1			1
*Lophopedia pygmaea*	s	8			8
Melipona eburnea *	s	5	1	8	14
Melipona nigrescens *	s	9	2	2	13
*Melissodes (Ecplectica) tintinnans*	g			14	14
*Monoeca* sp. Nov.	g			1	1
Nannotrigona melanocera *	s	18	17		35
Oxytrigona mulfordi *	s	66			66
Oxytrigona obscura *	s	23			23
*Paratetrapedia connexa*	s	1			1
*Paratetrapedia flaveola*	s	1	3		4
*Paratetrapedia lugubris*	s		1		1
*Paratetrapedia nr. Vogeli*	s	2			2
*Paratetrapedia vogeli*	s	2			2
Paratrigona pacifica *	o	2	25		27
Partamona epiphytophila *	o	5	2	1	8
Partamona epiphytophila? *	o		1		1
Partamona testacea *	g		15		15
*Plebeia* cf. kerri *	g,s,o		18		18
*Plebeia* sp. 1 *	g,s,o	36			36
*Plebeia* sp. 2 *	g,s,o	2	6		8
*Plebeia* sp. 3 *	g,s,o		1		1
Ptilotrigona lurida *	s	4	6		10
Ptilotrigona pereneae *	s		1	5	6
*Scaptotrigona* sp. 1 *	s		11		11
Scaptotrigona tricolorata *	s			1	1
Scaura latitarsis *	o	38	6		44
Scaura longula *	s		11		11
Tetragona dissecta *	s	1	42	1	44
Tetragona goettei *	s	1	4	5	10
Tetragonisca angustula *	s,o	12	18	1	31
*Thygater analis*	g		2		2
Trigona amalthea *	g,s,o	46	31	22	99
Trigona amazonensis *	g,s,o	41	47		88
*Trigona* cf. crassipes *	g,s,o	1	2		3
Trigona cilipes *	g,s,o		1		1
Trigona crassipes *	g,s,o	1	6		7
Trigona dimidiata *	g,s,o			1	1
Trigona fuscipennis s.l. *	g,s,o	13	35	73	121
Trigona guianae *	g,s,o	18	9		27
Trigona hypogea *	g,s,o	2	1		3
Trigona lacteipennis *	g,s,o	1		1	2
Trigona pallens *	g,s,o	48	24		72
Trigona recursa *	g,s,o	27	5		32
Trigona truculenta *	g,s,o	4			4
*Trigonisca* sp. 1 *	s		2		2
*Xylocopa (Neoxylocopa) andica*	s		3		3
*Xylocopa (Neoxylocopa) frontalis*	s		5		5
*Xylocopa (Neoxylocopa) tegulata*	s	3	1		4
**Colletidae (S = 8)**		**29**			**29**
*Colletes* cf. *rugicollis*	g,s	9			9
*Hylaeus* sp. 1	g,s,o	1			1
*Hylaeus* sp. 2	g,s,o	9			9
*Hylaeus* sp. 3	g,s,o	4			4
*Hylaeus* sp. 4	g,s,o	3			3
*Hylaeus* sp. 5	g,s,o	1			1
*Hylaeus* sp. 6	g,s,o	1			1
*Ptiloglossa* sp.	g	1			1
**Halictidae (S = 72)**		**392**	**129**	**24**	**545**
*Augochlora pachytes*	g	1			1
*Augochlora* sp. 1	g,s	18	3	2	23
*Augochlora* sp. 2	g,s		2		2
*Augochlora* sp. 3	g,s	2			2
*Augochlora* sp. 4	g,s	1			1
*Augochlora* sp. 5	g,s		1	1	2
*Augochlora* sp. 6	g,s	1			1
*Augochlora* sp. 7	g,s		1		1
*Augochlora* sp. 8	g,s	1	1		2
*Augochlora* sp. 9	g,s		4		4
*Augochlora* sp. 10	g,s		2		2
*Augochlora* sp. 11	g,s		2		2
*Augochlora* sp. 12	g,s		2		2
*Augochlora* sp. 13	g,s	1			1
*Augochlora* sp. 14	g,s		1		1
*Augochlora* sp. 15	g,s		1		1
*Augochloropsis* sp. 1	g	6			6
*Augochloropsis* sp. 2	g	2			2
*Augochloropsis* sp. 3	g	1	1		2
*Augochloropsis* sp. 4	g	7			7
*Augochloropsis* sp. 5	g		3		3
*Augochloropsis* sp. 6	g	7	4		11
*Augochloropsis* sp. 7	g	1	3		4
*Augochloropsis* sp. 8	g		2	1	3
*Augochloropsis* sp. 9	g		3		3
*Augochloropsis* sp. 10	g		5		5
*Augochloropsis* sp. 11	g		1		1
*Augochloropsis* sp. 12	g	1	1		2
*Augochloropsis* sp. 13	g	1	1	2	4
*Caenohalictus* sp.	g			1	1
*Dialictus* sp. 1	g,s	5	4	1	10
*Dialictus* sp. 2	g,s	5			5
*Dialictus* sp. 3	g,s	20	4	1	25
*Dialictus* sp. 4	g,s	1	3	5	9
*Dialictus* spp.	g,s	1			1
*Habralictus* sp.	g	3	1		4
*Habralictus* sp. 1	g	10			10
*Habralictus* sp. 2	g	43	1		44
*Habralictus* sp. 3	g	5	2		7
*Habralictus* sp. 4	g	1			1
*Megalopta* sp. 1	s			2	2
*Megaloptina* sp.	g	1			1
*Neocorynura* sp. 1	g,s	7			7
*Neocorynura* sp. 2	g,s	4	3		7
*Neocorynura* sp. 3	g,s	1	4		5
*Neocorynura* sp. 4	g,s	14	1		15
*Neocorynura* sp. 5	g,s		5		5
*Neocorynura* sp. 6	g,s	4	2	1	7
*Neocorynura* sp. 7	g,s	2			2
*Neocorynura* sp. 8	g,s	1			1
*Neocorynura* sp. 9	g,s	1			1
*Neocorynura* sp. 10	g,s	3	2		5
*Neocorynura* sp. 11	g,s		5		5
*Neocorynura* sp. 12	g,s	1			1
*Neocorynura* sp. 13	g,s	1			1
*Neocorynura* sp. 14	g,s	1			1
*Neocorynura* sp. 15	g,s	1			1
*Neocorynura* sp. 16	g,s	7			7
*Neocorynura* sp. 17	g,s		1		1
*Neocorynura* sp. 18	g,s	4			4
*Neocorynura* sp. 19	g,s	1	1		2
*Neocorynura* sp. 20	g,s		1		1
*Neocorynura* sp. 21	g,s	1			1
*Neocorynura* sp. 22	g,s		1		1
*Neocorynura* sp. 23	g,s	1			1
*Neocorynura* sp. 24	g,s		1		1
*Neocorynura* sp. 25	g,s		4		4
*Neocorynura* sp. 26	g,s	1			1
*Neocorynura* sp. 27	g,s	10			10
*Pereirapis semiaurata*	g	155	31	5	191
*Pseudaugochlora graminea*	g	25	8		33
*Temnosoma* sp. Nov.	cp			2	2
**Megachilidae (S = 8)**			**10**	**1**	**11**
*Coelioxys (Acrocoelioxys) tolteca*	cp		1		1
*Coelioxys (Rhinocoelioxys) platygnatha*	cp		1		1
*Megachile (Acentron)* sp. 1	g,s,o		1		1
*Megachile (Leptorachis)* sp. 1	g,s,o			1	1
*Megachile (Leptorachis)* sp. 2	g,s,o		1		1
*Megachile (Leptorachis)* sp. 3	g,s,o		1		1
*Megachile (Moureapis)* sp. 1	g,s,o		3		3
*Megachile (Pseudocentron)* sp. 1	g,s,o		2		2
**Total**		972	595	229	1796
**Richness (S)**		116	109	43	181
**Shannon (H’)**		0.00	0.00	0.00	0.00
**Evenness**		0.00	0.00	0.00	0.00

**Table 2:** Similarity coefficient of pairwise site comparisons at three sites in Alto Mayo, Peru; RN: Río Negro, RA: Ríos Aguas Claras-Amangay, TI: Tingana.

Paired Site Comparisons	Coefficient of Jaccard (Cj)	Coefficient of Sørensen (Cs)
RN x RA	0.25	0.56
RN x TI	0.15	0.29
RA x TI	0.18	0.41

**Table 3:** Selected comparison of species reported here with those previously reported from San Martín. Detailed comparison with number of species reported previously, number of species in this study and, finally, the number of species found in both studies. Bumble bees or Bombini are records from across the country, including San Martin [23]; orchid bees or Euglossini are records from lower and similar elevation sites closer to lowland rainforest [16]; stingless bees or Meliponini are records from a similar elevation but a mountain ridge closer to lowland rainforest [21].

	Number of Species from Literature	Number of Species in Present Study (Unique)	Species Found in Both Studies
Bombini	3	3 (1)	2
Euglossini	41	20 (5)	15
Meliponini	51	38 (21)	17
**TOTAL**	95	61 (27)	34

## Data Availability

Data can be requested from the authors.
